# Right bundle branch block pattern during right ventricular permanent pacing: Is it safe or not?

**Published:** 2007-08-01

**Authors:** Okan Erdogan, Feyza Aksu

**Affiliations:** Trakya Universitesi Tip Fakultesi, Kardiyoloji Edirne 22030 Turkey

**Keywords:** cardiac pacing, malposition, right bundle branch block, complication, electrocardiography

## Abstract

The present case report describes a patient with dual chamber pacemaker whose surface ECG demonstrated paced right bundle branch block pattern suggesting a malpositioned ventricular lead in the left ventricle. However, diagnostic work-up revealed that the lead was appropriately located in the right ventricular apex. Diagnostic maneuvers and clues for differentiating safe right bundle branch block pattern during permanent pacing are thoroughly revisited and discussed within the article.

An 81-year-old woman with history of hypertension and diabetes mellitus underwent dual chamber permanent pacemaker implantation for symptomatic high degree atrioventricular block two years ago. Since then she was asymptomatic and her surface ECG showed atrial tracking and ventricular pacing with an unusual right bundle branch block (BBB) configuration. QRS axis on the frontal plane was around -60º. V1 and V2 showed Rs and R complexes, respectively. Precordial transition occurred by lead V3 (Figure 1a). Upon suspicion of a possible malpositioned lead in the left ventricle we took another surface ECG by placing the precordial leads one interspace lower than standard that surprisingly eliminated right BBB appearance and resulted in the inscription of deep QS complexes in V1 and V2 (Figure 1b). In addition, transthoracic echocardiography clearly showed the pacing lead traversing from the right atrium to the right ventricle and lying in the right ventricular apex (Figure 2). Fluoroscopic views taken on both directions also revealed right sided and antero-inferiorly oriented ventricular pacing lead whose tip was positioned infero-apically in the right ventricular apex (Figure 3aand 3b). After confirming the correct lead position in the right ventricular apex with the aid of echocardiography and fluoroscopy, the patient was reassured that everything was fine with her pacemaker system.

## Discussion

Transvenous right ventricular pacing usually demonstrates left BBB pattern. Paced right BBB pattern may represent coronary venous pacing, septal or free wall perforation and left ventricular pacing through retrograde transarterial route or intracardiac defects such as patent foramen ovale, ventricular septal defect [1-3]. However, uncomplicated pacing even in the right ventricle may sometimes produce right BBB type paced QRS complexes [4]. Therefore, it is clinically important to determine whether a right BBB pattern induced by ventricular pacing is the result of a malpositioned lead or uncomplicated transvenous right ventricular pacing. Positioning the ventricular lead inadvertently in the left ventricle may create serious clinical problems and require life long anticoagulation or lead extraction. Fortunately, not all right BBB configuration is associated with left ventricular pacing. Most will have right ventricular leads in appropriate positions. Klein et al. [5] reported eight patients with right BBB patterns in leads V1-2, left BBB pattern in lead I, and pacing leads located in the right ventricular apex. They recognized that placement of leads V1-2 one interspace lower than standard could eliminate right BBB appearance. Coman et al. [6] reported seven cases with right BBB pattern during permanent right ventricular pacing. Placing the leads V1-2 one interspace lower than standard resulted in disappearance of right BBB morphology and inscription of QS or rS complexes in V1-2. Each patient had leads located in the distal right ventricular septum or apex. However, four patients whose right BBB pattern could not be eliminated by moving of leads V1-2 had their pacing leads located in the mid septum. Hence, they suggested that this technique reliably distinguished patients with midseptal leads from those with leads in the distal septum and apex. In our patient moving the leads V1-2 one interspace lower than standard also eliminated right  BBB pattern and resulted in QS complexes in V1-2. We also confirmed with the aid of echocardiography and fluoroscopy that the tip of the ventricular lead was located in the right ventricular apex. Coman et al. [6] developed an algorithm to differentiate right and left ventricular right BBB pacing morphologies using frontal axis and precordial transition. They suggested that after excluding left ventricular pacing from the proximal and mid septum, a frontal axis of 0º to -90º and precordial transition by V3 may be able to differentiate uncomplicated right ventricular septal or apical pacing from all other forms of  left ventricular pacing with 86% sensitivity, 99% specificity, and 95% positive predictive value. The frontal plane axis found in our patient was around -60º and precordial transition occurred by V3 which was suggestive of uncomplicated right ventricular apical pacing according to the above mentioned algorithm. Together with this finding and elimination of right BBB pattern by moving the leads V1-2, as previously described, we satisfactorily determined safe right ventricular pacing in our patient. We also confirmed the correct lead position by echocardiography and fluoroscopy. Several hypotheses were proposed, as to the mechanism why right BBB configuration occurs in normally placed right ventricular leads. One plausible mechanism proposed by Mower et al. [7] was that portions of the interventricular septum which are anatomically right ventricle may behave functionally and electrically as left ventricle. Another suggestion made by Barold et al [8] was that the right BBB pattern could be the result of a combination of right ventricular activation delay due to severe disease of the right ventricular conduction system and early penetration of the electrical impulse into the left ventricular conduction system.

## Conclusion

Right BBB pattern during ventricular pacing does not always point out left ventricular pacing due to a malpositioned ventricular lead. Safe uncomplicated right BBB pattern may be seen with correctly positioned right ventricular leads in the distal septum or apex. Simple techniques such as moving the leads V1-2 one interspace lower than standard or combining frontal axis and precordial transition as an algorithmic approach may correctly identify the correct position of the lead. Echocardiography and fluoroscopy are also useful confirmatory techniques that are inevitable in cases of doubt.

## Figures and Tables

**Figure 1:**
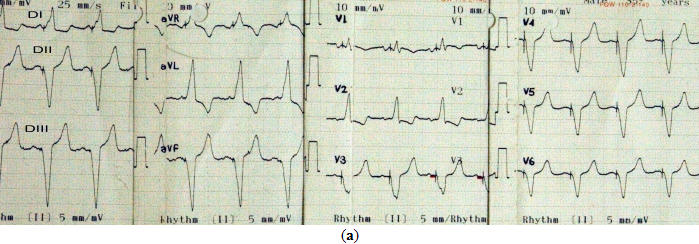

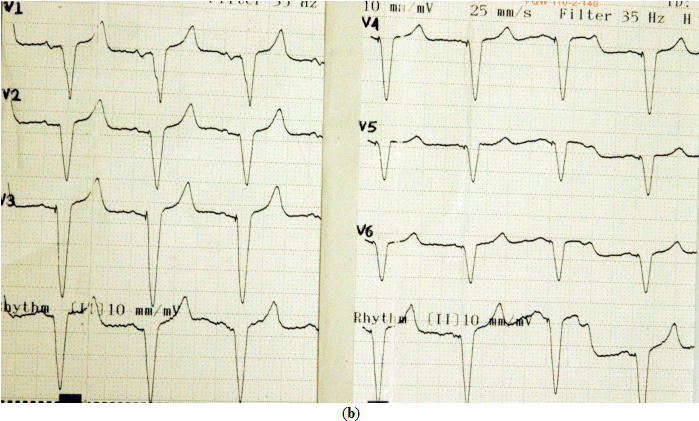
**(a)** Surface ECG showing atrial tracking and ventricular pacing with right BBB pattern in V1 and V2 (Rs and R complexes) associated with a frontal axis of  -60º.  **(b)** Moving the leads V1-2 one interspace lower than standard results in left BBB configuration with the inscription of QS complexes in leads V1 and V2.

**Figure 2:**
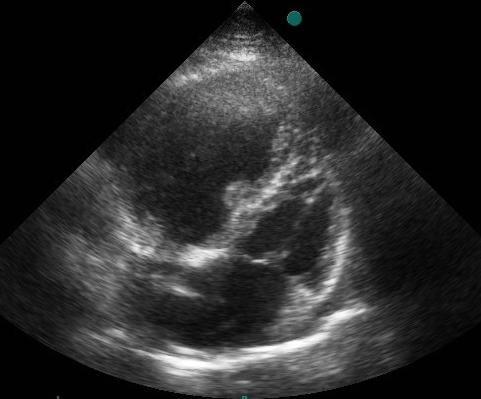
Parasternal transthoracic echocardiographic view demonstrating and confirming the ventricular pacing lead going from the right atrium to the right ventricle with its tip located in the apical position.

**Figure 3:**
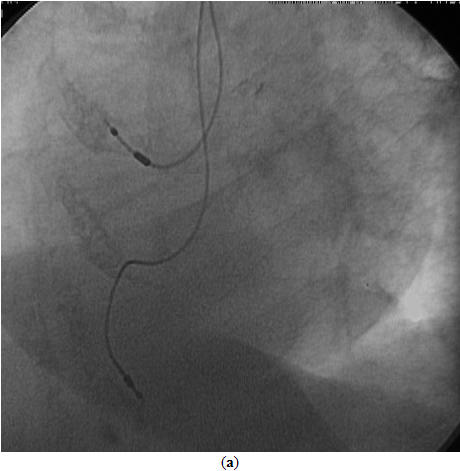

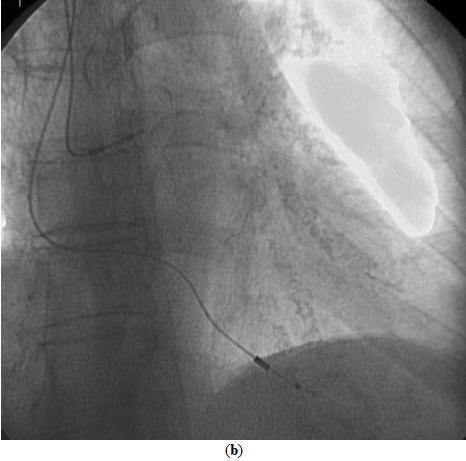
**(a)** Fluoroscopic view (45º left anterior oblique) nicely shows both atrial and ventricular leads on the right side of the septum. The tip of the ventricular lead is fixed in the distal infero-apical position of the right ventricular apex.  **(b)** Fluoroscopic view (30º right anterior oblique) shows the ventricular pacing lead oriented anteriorly and inferiorly in the right ventricular apex.
